# Public acceptance of genome editing in Saudi Arabia is driven by therapeutic benefit despite limited awareness

**DOI:** 10.3389/fmed.2026.1827655

**Published:** 2026-06-25

**Authors:** Hala Aldahshan, Tourkiah Alessa, Tahani O. Alkahtani, Abeer Al-Hubaysh, Hamood AlSudais, Sana Saeed Alqarni, Nahla Bakhamis

**Affiliations:** 1 Department of Clinical Laboratory Sciences, College of Applied Medical Sciences, King Saud University, Riyadh, Saudi Arabia; 2 Department of Biomedical Technology, College of Applied Medical Sciences, King Saud University, Riyadh, Saudi Arabia; 3 Department of Radiological Sciences, College of Health and Rehabilitation Sciences, Princess Nourah Bint Abdulrahman University, Riyadh, Saudi Arabia; 4 Department of Biology, College of Science, Princess Nourah Bint Abdulrahman University, Riyadh, Saudi Arabia

**Keywords:** bioethics, CRISPR technologies, ethical acceptance, genome editing, human genomics, public awareness

## Abstract

Genome editing technologies are transforming precision medicine and rare disease therapeutics; however, their responsible clinical integration depends on public awareness, ethical acceptance, and trust in regulatory oversight. Evidence from culturally diverse populations remains limited, particularly in regions undergoing rapid investment in genomic medicine. A large nationwide cross-sectional survey was conducted using a structured, self-administered electronic questionnaire distributed to adults aged 18 years and older. The instrument assessed demographic characteristics, awareness of genome editing, perceived benefits and risks, ethical concerns, and acceptance of genome editing applications. Descriptive statistics, correlation analyses, and regression analyses were performed to examine factors associated with acceptance. A total of 1,712 participants completed the survey. Fewer than half of respondents reported prior awareness of genome editing, indicating limited baseline familiarity. Support for therapeutic applications was high, particularly when interventions were framed as treatments for serious or life-threatening conditions. Favourable attitudes and perceived clinical benefit were strongly associated with acceptance, while perceived risk demonstrated a modest negative association. Greater awareness was associated with increased ethical caution. Trust in national regulatory frameworks significantly strengthened confidence in gene therapy applications. These findings indicate a relatively supportive public perspective toward therapeutic genome editing in Saudi Arabia, particularly when its use is framed within appropriate ethical and regulatory boundaries. By providing population level evidence from an underrepresented region undergoing rapid genomic development, this study offers insights that may inform culturally responsive policy, public engagement, and responsible clinical translation of genome editing technologies.

## 1 Introduction

Genome editing technologies, particularly CRISPR/Cas9, have enabled precise DNA modification in living cells, catalyzing rapid clinical translation (1). Several gene therapy products have now received regulatory approval, with extensive pipelines targeting cancers, hereditary disorders, and other serious conditions (2). However, these advances have also raised profound ethical, social, and regulatory challenges, particularly regarding safety, equity, and public oversight in clinical implementation (3).

International scientific and ethics bodies increasingly emphasize that genome editing governance must be grounded in public values and shaped through meaningful societal engagement (4). Public discourse fundamentally influences ethical and regulatory frameworks governing genomic technologies, with deliberative processes translating societal values into actionable policy recommendations (5, 6). This process is particularly critical in regions with distinct cultural and religious contexts that shape moral reasoning about genetic intervention. Understanding public attitudes toward genome editing is key in the development of legitimate and effective governance frameworks.

Cross cultural research consistently reveals strong public support for genome editing when applied to the treatment or prevention of serious diseases. Large scale surveys conducted across North America, Europe, and East Asia, have consistently demonstrated broad public acceptance of therapeutic genome editing, particularly for life threatening or genetically inherited conditions (7–10). Systematic reviews have identified key determinants of this support, including perceived medical necessity, expected benefit risk balance, scientific literacy, and trust in regulatory systems (11, 12). Despite these global insights, the empirical literature on public attitudes toward genome editing remains geographically concentrated in North America, Europe, and East Asia (11) with limited empirical data available from countries such as Saudi Arabia (13), particularly from general population-based studies.

The Middle East represents a particularly relevant setting for examining public perspectives on genome editing due to the intersection of medical need, sociocultural context, and rapid genomic development. Several countries in the region, including Saudi Arabia, experience elevated prevalence of autosomal recessive disorders, partly associated with consanguineous marriage patterns, creating a substantial clinical burden that could potentially benefit from genomic interventions (14, 15). At the same time, Saudi Arabia has made significant national investments in genomic medicine initiatives, including the Saudi Human Genome Program (SHGP), positioning the country as an emerging regional leader in precision medicine (16). These factors make public attitudes toward genome editing especially consequential for future clinical adoption and governance.

While some research has explored the views of healthcare professionals (13), comprehensive data from the general population remains scarce. The scarce data limits both national policy formation and international comparative analyses. Capturing these perspectives is, therefore, critical for developing equitable, culturally informed pathways for genome editing adoption.

In the present study, genome editing is discussed primarily in the context of therapeutic somatic applications aimed at treating or preventing serious diseases, rather than germline modification. Although CRISPR-based systems represent the most widely recognized platform, the survey addressed genome editing concepts broadly to capture public perceptions of clinical genetic interventions.

This research provides a comprehensive assessment of knowledge, attitudes, and perception concerns regarding genome editing among Saudi adults. Specifically, it examines the (1) public awareness and understanding of genome editing and gene therapy, (2) acceptance of genome editing applications, and (3) demographic and psychosocial association of acceptance. By generating empirical evidence from an underrepresented region undergoing rapid genomic development, this research provides timely insights that may inform public engagement strategies, culturally sensitive policy considerations, and responsible clinical translation of genome editing technologies.

## 2 Methods

This study employed a cross-sectional survey design to assess public awareness, attitudes, and acceptance of genome editing technologies in Saudi Arabia. The survey targeted adult residents aged 18 years and above across all regions of the Kingdom. An online distribution approach was used to facilitate broad demographic reach. Participation was voluntary, anonymous, and preceded by electronic informed consent.

To determine the appropriate sample size, we used a standard formula using Raosoft, Inc. (Seattle, WA, United States) for estimating proportions in large populations assuming a 50% response distribution. Based on a total Saudi population of approximately 35 million, a minimum sample of 384 respondents would be required to achieve a 95% confidence level with a ± 5% margin of error.

The questionnaire was adapted from previously validated international surveys on genome editing perceptions (7, 14, 15). It was comprised of five main sections: demographic and health related variables (7 items), knowledge (6 items), acceptability (10 items), attitudes (5 items), and influencing factors (15 items). Demographic variables included age, gender, marital status, education level, region of residence, and occupation.

Knowledge items were measured using dichotomous and Likert type responses, with scores converted into percentage values. Acceptability and attitudes were assessed using a 5-point Likert scale ranging from 1 (strongly disagree) to 5 (strongly agree), where higher scores reflected greater acceptance and more positive attitudes toward genome editing technologies. Influencing factors and demographic variables were analyzed descriptively.

Internal consistency reliability was assessed using Cronbach’s alpha coefficient. The knowledge and acceptability constructs demonstrated good reliability (*α* = 0.87 and α = 0.86, respectively), while the attitudes construct showed acceptable reliability (α = 0.78).

The content validity was evaluated by a panel of subject matter experts (*n* = 6) with expertise in genetics, bioethics, and public health using Item-Level Content Validity Indices and a 4- point rating Scale-Level Content Validity Indices. The Scale-Level Content Validity Index using Universal Agreement (S-CVI/UA) was 0.92, and the Scale-Level Content Validity Index based on the Average of Item-Level Content Validity Indices (S-CVI/Ave) was 0.99, demonstrating excellent content validity. Based on their feedback, modifications were made to improve clarity, cultural appropriateness, and conceptual alignment with the Saudi context.

A pilot study was also conducted with a sample of 20 participants from the target population (who were excluded from the final analysis) to evaluate face validity, item clarity, and response burden. Based on pilot responses, minor revisions were made to the question structure and language. Final data collection was conducted via an online survey platform from March 29, 2025 to June 29, 2025, with participants recruited through social media platforms, institutional mailing lists, and professional networks.

### 2.1 Statistical analysis

Descriptive statistics (frequencies, percentages, means, and standard deviations) were used to summarize participants’ socio-demographic characteristics and responses. Internal consistency of key subscales was assessed using Cronbach’s alpha, with all subscales demonstrating acceptable reliability (*α* > 0.70). Correlation analyses (Pearson’s *r*) were conducted to examine relationships between attitudinal constructs. Strong correlations were observed among the therapeutic attitude items, suggesting potential multicollinearity. Variance Inflation Factor (VIF) values were also examined and confirmed the presence of multicollinearity. According to Hair et al. (16), VIF values between 5 and 10 indicate significant multicollinearity. This violates the assumptions required for multivariable regression analysis. Therefore, multivariable regression was not performed. Instead, a simple linear regression analysis was conducted to examine the association between therapeutic attitude (independent variable) and genome editing acceptability (dependent variable). All analyses were performed using SPSS v29, with significance set at *p* < 0.05 (two-tailed). Given the conceptual overlap between therapeutic attitude and acceptability constructs, the regression analysis was interpreted as exploratory rather than predictive.

### 2.2 Ethical consideration

The study protocol was approved by the Institutional Review Board of King Saud University (IRB no: KSU-HE-25-131). All procedures adhered to the Declaration of Helsinki principles and Saudi Arabian research ethics guidelines. Participants provided informed consent electronically, and all data were collected and stored in accordance with international data protection standards.

## 3 Results

### 3.1 Socio-demographic characteristics

A total of 1,712 participants completed the survey and were included in the final analysis. Table 1 summarizes the socio-demographic characteristics of the respondents. The sample was predominantly female (60.5%) and largely composed of younger adults, with over 63% aged between 18 and 39 years. Educational attainment was high, with over 80% of participants holding a bachelor’s degree or higher. More than half of the sample (56.3%) resided in the Central Region, and 26.1% identified as medical professionals. Notably, over 50% reported having a family member affected by cancer, and 27.3% reported a personal or familial experience with inherited disorders. These characteristics provide important context for interpreting subsequent results on awareness, attitudes, and acceptance of genome editing technologies.

**Table 1:** Socio-demographic characteristics of study participants (*N* = 1,712).

Characteristic	*N*	%
Nationality		
Saudi	1,598	93.3%
Other	114	6.7%
Gender		
Male	676	39.5%
Female	1,036	60.5%
Age		
18–29	529	30.9%
30–39	554	32.4%
40–49	425	24.8%
50–59	141	8.2%
60 or older	63	3.7%
Region		
Central region	964	56.3%
Northern region	160	9.3%
Eastern region	180	10.5%
Western region	266	15.5%
Southern region	142	8.3%
Marital status		
Single	630	36.8%
Married	987	57.7%
Widowed	11	0.6%
Divorced	84	4.9%
Parental status		
Yes	931	54.4%
No	781	45.6%
Qualification		
Intermediate school	13	0.8%
High school	179	10.5%
Diploma	119	7.0%
University or college (bachelor’s)	760	44.4%
Master’s degree	400	23.4%
PhD	223	13.0%
Other	18	1.1%
Occupation^a^		
Medical professional	447	26.1%
Academic	354	20.7%
Student	319	18.6%
No profession	232	13.6%
Other	516	30.1%
Cancer		
Patient with cancer	41	2.4%
Related to patient with cancer	866	50.6%
Both	18	1.1%
None	787	46.0%
Inherited disorder		
Patient with an inherited disorder	151	8.8%
Related to a patient with an inherited disorder	316	18.5%
Both	52	3.0%
None	1,193	69.7%

a Respondents were allowed to choose more than one option.

### 3.2 Genome editing awareness and knowledge

Public awareness of genome editing was moderate across the sample. As shown in Table 2, 46% of respondents reported prior familiarity with the concept. Awareness of regulatory developments was notably lower; only 32.7% of participants were aware that the Saudi Food and Drug Authority (SFDA) had approved gene therapy for treating inherited diseases. However, the potential for building trust through policy visibility was evident as nearly 90% of respondents indicated that such regulatory endorsements increased their confidence in gene therapy to at least a moderate extent (see Table 3).

**Table 2:** Public awareness and knowledge regarding genome editing technologies.

Item	N	%^a^
Have heard about genome editing		
Yes	787	46%
No	925	54%
Familiar that SFDA has approved gene therapy for treating inherited diseases		
Yes	560	32.7%
No	1,152	67.3%
Impact of SFDA’s decisions on trust in gene therapy		
None at all	171	10.0%
Slightly	144	8.4%
A moderate amount	343	20.0%
Very much	674	39.4%
Significantly	380	22.2%
Source of information (for those who have heard)		
Healthcare provider	227	28.8%
Internet	479	60.9%
Friend or family member	111	14.1%
Articles or book	288	36.6%
University course	235	29.9%
Training and workshop	146	18.6%
How would you describe genome editing? (for respondents who had previously heard of the term)		
A method to alter the DNA of a living organism	537	68.2%
A technique for cloning organisms	27	3.4%
A process used to sequence DNA	164	20.8%
None of the above	59	7.5%
How confident in understanding genome editing (for those who have heard)		
Not confident	109	13.9%
Slightly confident	172	21.9%
Moderately confident	292	37.1%
Confident	151	19.2%
Very confident	63	8.0%
Understanding of the explanation about genome editing (for those who never heard)		
I understood it completely	399	43.1%
I understood it for the most parts	367	39.7%
I did not understand it very well	130	14.1%
I did not understand it at all	29	3.1%

^a^Percentages are presented only for valid cases.

**Table 3:** Model summary for the association between therapeutic attitude and therapeutic acceptability.

Model	R	R Square	Adjusted R square	Std. error of the estimate
1	0.899^a^	0.808	0.808	5.883

a Predictors: (constant), therapeutic attitude.

Among those aware of genome editing (*n* = 787), knowledge accuracy was mixed. When asked to identify the correct definition, 68.2% selected “a method to alter the DNA of a living organism.” However, 20.8% confused it with DNA sequencing, 3.4% selected cloning, and 7.5% responded “none of the above.” Confidence in understanding varied with 64.2% reported feeling moderately to highly confident, while 35.8% expressed low or no confidence. Sources of information were primarily digital or academic, with the internet (60.9%) and scientific materials (36.6%) most cited, while healthcare providers played a comparatively minor role (28.8%).

For the 925 respondents who were initially unaware of genome editing, a brief explanatory infographic was presented during the survey (Supplementary Figure 1). Following this intervention, the majority (82.8%) reported that they understood the concept completely or for the most part, indicating strong receptiveness to targeted visual education even among previously uninformed individuals.

### 3.3 Ethical acceptance toward therapeutic use

Participants demonstrated strong support for the use of genome editing in therapeutic contexts. The highest levels of agreement were observed when editing was framed as a tool to eliminate the root cause of a disease, with 78.2% expressing agreement or strong agreement. Similarly, 71.6% supported its use to improve symptoms, even if not curative.

**Figure 1:**
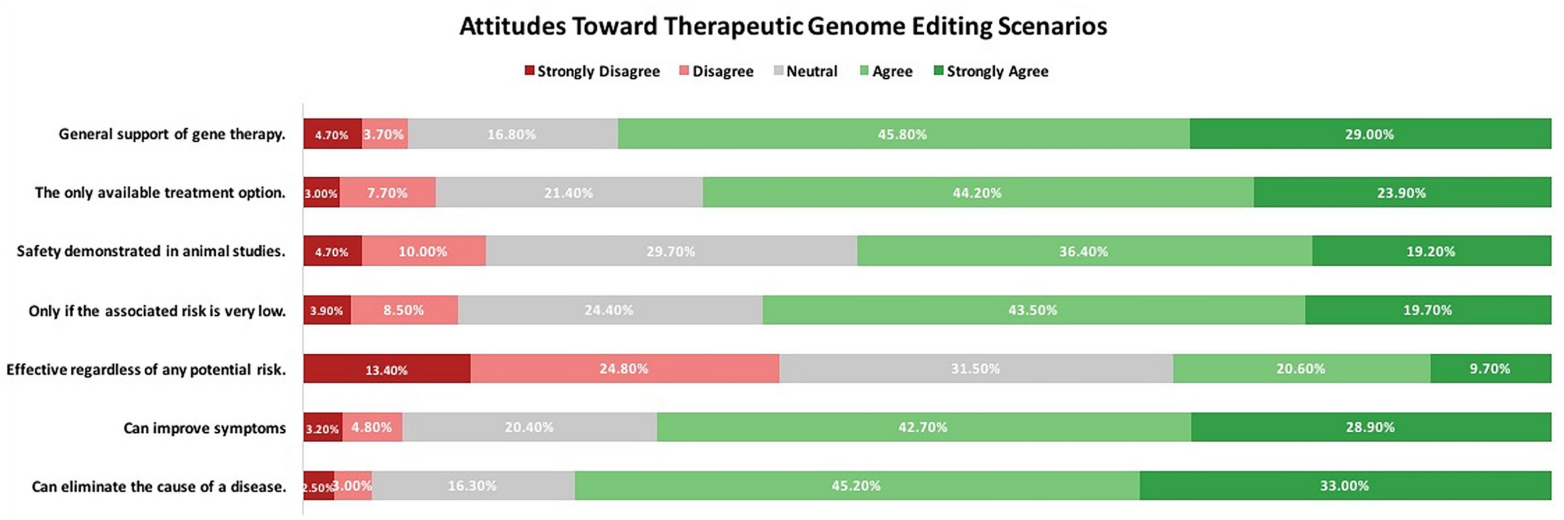
Public attitudes toward therapeutic genome editing applications. Distribution of participant responses regarding therapeutic uses of genome editing, including disease treatment, symptom improvement, and use in life-threatening conditions. Responses were measured using a 5-point Likert scale ranging from strongly disagree to strongly agree.

Genome editing was also widely supported when framed as the only available treatment option, with 68.1% in agreement. Acceptability remained relatively high under conservative clinical scenarios, such as when the associated risk is very low (63.2% agreement) or when safety has been demonstrated in animal studies (55.6%). These patterns indicate that respondents are not uniformly permissive but instead calibrate their support according to perceived necessity and safety assurances. General support for gene therapy was also favorable, with 74.8% of those who responded to this item expressing a positive or strongly positive attitude (Figure 1). Given the conceptual similarity among these items, they were treated as a unified therapeutic attitude construct in subsequent analyses.

### 3.4 Acceptability of genome editing applications

**Figure 2:**
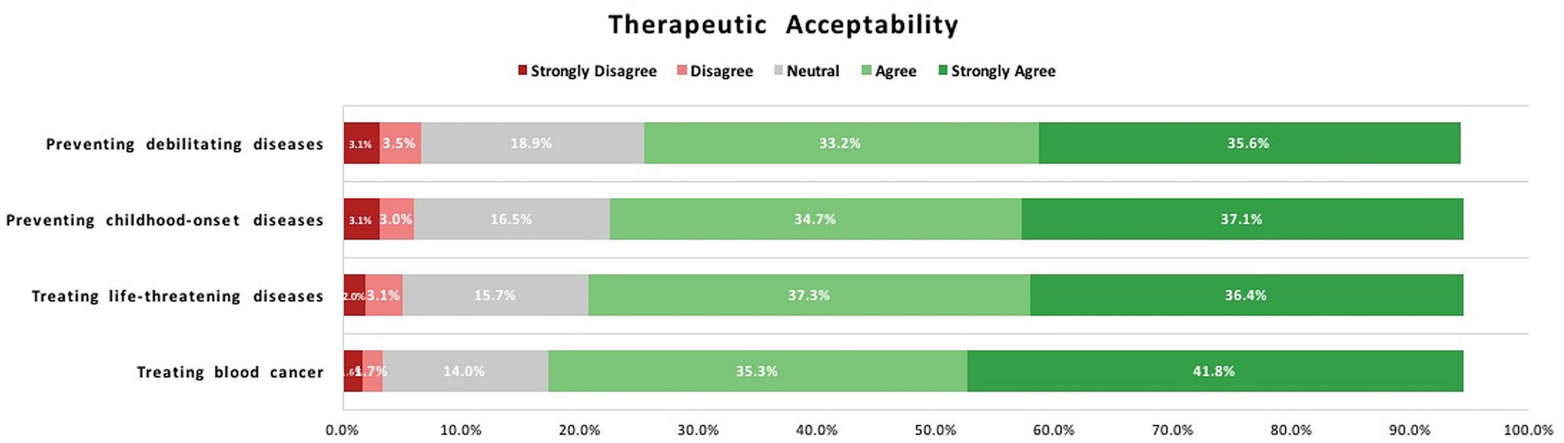
Public acceptance of therapeutic genome editing applications in different clinical scenarios. Participant responses assessing acceptance of genome editing for conditions such as blood cancer, life-threatening diseases, and inherited disorders. Percentages represent combined Likert scale responses across levels of agreement.

Respondents demonstrated high levels of support for genome editing when used in therapeutic contexts. As shown in Figure 2, over 80% of participants agreed or strongly agreed with genome editing to treat blood cancer in patients lacking other treatment options. Similarly, 77.7% supported its use in children or adults with life-threatening diseases, and 75.5% favored its use to prevent childhood-onset life-threatening conditions. Acceptance was slightly lower, but still substantial, for preventing debilitating diseases, with 72.1% expressing agreement or strong agreement. Detailed response distributions are provided in Supplementary Table 1.

#### 3.4.1 Factors associated with genome editing acceptability

Preliminary analyses showed strong correlations among therapeutic attitude variables, which violated the assumptions required for multivariable regression analysis. Therefore, a simple linear regression analysis was conducted to examine the association between therapeutic attitude and genome editing acceptability. The model was highly significant (*F*(1,1710) = 7219.36, *p* < 0.001) and demonstrated a strong association with genome editing acceptability (R^2^ = 0.808, Adjusted R^2^ = 0.808). Therapeutic attitude was strongly positively associated with genome editing acceptability (*B* = 0.959, *β* = 0.899, *p* < 0.001), indicating that more favourable attitudes toward therapy are associated with higher acceptability of genome editing (Table 4). The relatively high explained variance likely reflects conceptual overlap between the therapeutic attitude and acceptability constructs, which capture closely related evaluative dimensions.

**Table 4:** Coefficients for the association between therapeutic attitude and therapeutic acceptability^a^.

Model		Unstandardized coefficients		Standardized coefficients	t	Sig.
		B	Std. error	Beta		
1	(Constant)	−13.477	0.270		−49.894	0.000
	Therapeutic attitude	0.959	0.011	0.899	84.967	0.001

a Dependent variable: therapeutic acceptability.

**Figure 3:**
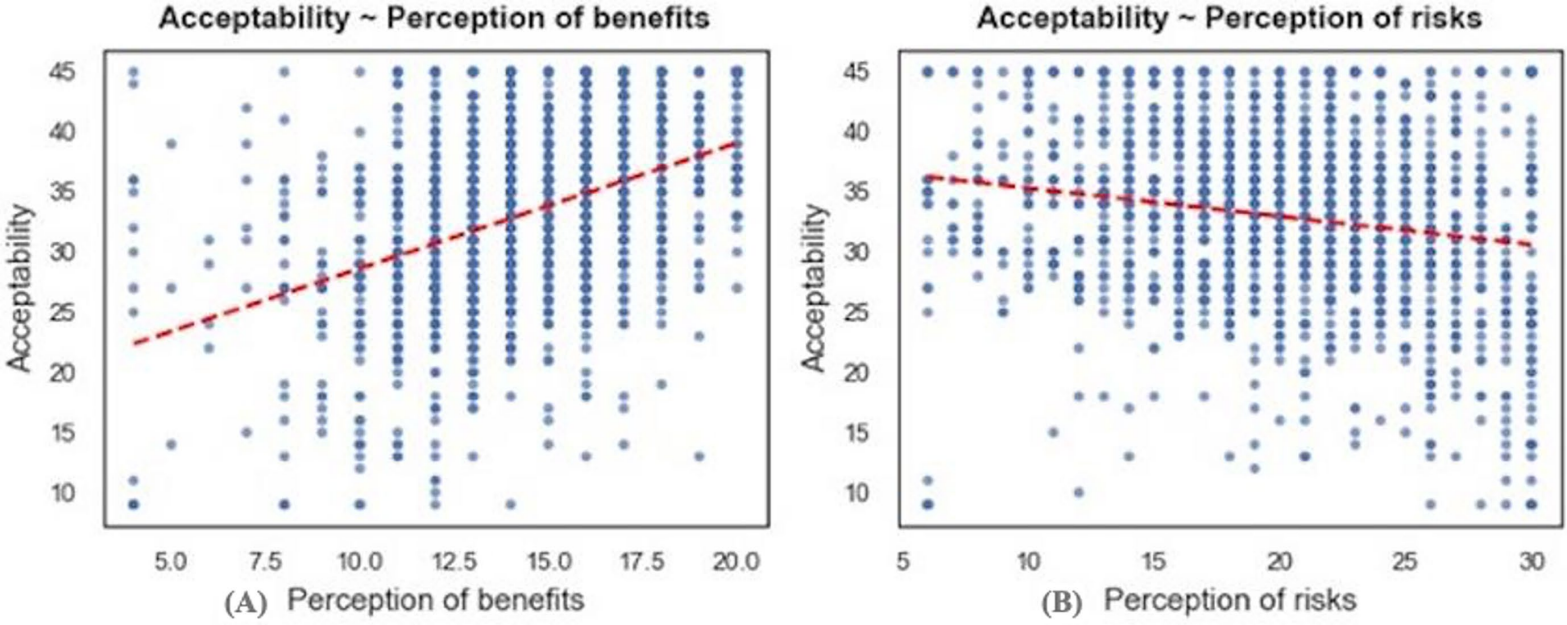
Associations between genome editing acceptability and psychological perception variables. Scatterplots with fitted regression lines illustrating the relationships between therapeutic acceptability scores and **(A)** perceived benefits and **(B)** perceived risks. Positive associations were observed for perceived benefits, whereas perceived risks showed a negative association with acceptability.

To explore psychological and perceptual factors associated with genome editing acceptability, unadjusted regression plots were generated (Figure 3). A positive association was observed between acceptability and perceived benefits (*β* = 0.519, *p* < 0.001). In contrast, perceived risks showed a negative association with acceptability (β = −0.149, *p* < 0.001). These findings suggest that positive therapeutic perceptions and perceived benefits are associated with higher acceptance of genome editing, whereas perceived risks are associated with lower acceptance.

## 4 Discussion

This study provides a large-scale assessment of public awareness and ethical acceptance of genome editing technologies in Saudi Arabia, offering insights from a culturally distinct region with growing investments in genomic medicine. Consistent with global patterns, Saudi respondents strongly supported therapeutic applications, particularly for life threatening or hereditary conditions. These results reinforce prior evidence that perceived medical necessity is a central driver of public acceptance across diverse populations.

Importantly, these findings contribute evidence from a region that remains underrepresented in the global literature on public attitudes toward genome editing. Examining public perceptions in diverse sociocultural contexts is essential for developing governance frameworks that are applicable across different healthcare systems, rather than being based solely on data from Western populations.

Public awareness of genome editing was present but not widespread, with fewer than half of respondents reporting prior familiarity with the concept. Awareness was higher among younger individuals, those with higher educational attainment, and healthcare professionals, consistent with international trends (17–19). However, deeper regulatory understanding remained limited, with only 32.7% aware that gene therapy is approved by the SFDA, reflecting a gap between general awareness and applied regulatory knowledge. Importantly, visibility of regulatory approval was strongly associated with increased trust, highlighting the role of transparent governance and clear institutional communication in strengthening public confidence.

Notably, prior knowledge of genome editing was associated with lower acceptance and attitude scores, representing one of the most policy relevant findings. This contrasts sharply with Western contexts, where familiarity typically predicts greater support (11, 20). Similar patterns have emerged in culturally conservative settings, where increased awareness may correlate with heightened ethical and regulatory skepticism. Jedwab et al. reported that individuals with professional genetics experience were less supportive of such interventions, reinforcing the idea that deeper knowledge may lead to greater caution rather than uncritical acceptance (21).

Several mechanisms may explain this inverse relationship. Media narratives in conservative settings often emphasize ethical breaches and safety risks, making informed individuals cautious (17). In addition, religious frameworks shape how knowledge is interpreted. For example, in Islamic contexts, gene editing may raise concerns regarding interference with natural creation or divine design (22, 23). Trust deficits may also moderate the relationship between knowledge and acceptance. Finally, limited bioethics education may produce technical understanding without ethical grounding (24), leaving informed individuals without a framework for integrating new knowledge into acceptance.

These findings demonstrate that familiarity does not automatically foster acceptance; rather, it may activate deeper moral scrutiny. However, the cross-sectional design of this study precludes causal inference regarding the direction of this relationship. Several alternative explanations must be considered. Individuals who actively seek information about genome editing may already hold more cautious or sceptical views, such that awareness reflects pre-existing moral concern rather than being caused by exposure to information. In addition, selective exposure to critical media narratives often emphasizing ethical risks or safety concerns may simultaneously influence both awareness and attitudes without a direct causal relationship between them. Reverse causation therefore cannot be excluded, as lower acceptance may itself motivate greater information-seeking behavior. To clarify the directionality of this relationship, longitudinal or experimental study designs, such as randomized knowledge provision interventions, would be required. These interpretive constraints are acknowledged as a limitation of the present study. Therefore, science communication strategies must integrate cultural, and ethical values alongside technical facts to foster meaningful public engagement.

Over 78% of respondents supported genome editing for treating diseases, with similarly high agreement for conditions such as blood cancer and other life-threatening diseases, reflecting the global pattern where medical necessity drives acceptance (8, 20). Prior exposure to serious disease, either personally or through affected relatives, was associated with higher acceptance of genome editing. This aligns with findings from oncology settings, where patients and caregivers often show greater openness to experimental therapies, driven by a sense of urgency and familiarity with the limitations of current treatments (25, 26). These experiences foster hope and elevate perceived necessity, underscoring the importance of patient centered narratives in shaping public trust and ethical policy engagement.

Regional variation in acceptance was also observed. Respondents from the Northern and Southern regions showed significantly different acceptance and attitude scores compared to those from the Central region. These differences may be partially explained by the distribution of professions and education levels in the sample, with fewer respondents from healthcare backgrounds in those regions. This aligns with previous findings that link genetic knowledge and attitudes to educational and professional background (13). Given Saudi Arabia’s geographic and cultural diversity, outreach campaigns should be regionally tailored to address local values and information ecosystems.

Psychological perceptions were closely associated with public acceptability of therapeutic genome editing. Greater perceived clinical benefit was positively associated with acceptance, whereas perceived risk showed a modest negative association. These findings are consistent with prior research in the United States, Australia, and Europe, where perceived benefit often outweighs risk concerns in shaping public attitudes toward emerging biotechnologies (7, 27–29). Notably, the strength of the benefit acceptance relationship in our study may reflect cultural values emphasizing therapeutic necessity and family centered care in Saudi Arabia. Conversely, the comparatively weaker impact of risk perception suggests that concerns around safety may be acknowledged, but do not strongly deter support if the potential medical benefits are clear.

These findings are particularly relevant in the context of healthcare systems undergoing rapid genomic development. Saudi Arabia’s national investments in genomic medicine initiatives like SHGP create an environment in which public perceptions may directly influence clinical adoption pathways. The strong association between regulatory trust and acceptance observed in this study reinforces international recommendations that responsible implementation of genome editing technologies should be accompanied by transparent oversight, inclusive dialogue, and culturally responsive communication (30). Inclusion of participants with varying levels of prior awareness reflects real world population diversity and allows evaluation of attitudes following brief informational exposure, which is highly relevant for designing public engagement interventions. Educational initiatives may benefit from integrating scientific information with ethical, cultural, and religious perspectives to support informed public dialogue rather than focusing solely on technical knowledge.

Situating these findings within a broader regional and international context strengthens their interpretive value. At the global level, a systematic review of 41 studies showed that public acceptance of gene therapy and genome editing is highest for medical applications, particularly for serious or fatal diseases, and is strongly influenced by perceived risk (31). Within the MENA region, a qualitative discussion involving researchers from multiple countries reported broad support for therapeutic genome editing alongside persistent ethical concerns regarding enhancement and misuse, highlighting the role of governance frameworks in shaping professional confidence (32).

These patterns are reflected in survey-based evidence from neighbouring contexts. In the United Arab Emirates, a cross-sectional survey reported moderate acceptance of genetic engineering (63%), with education level and prior scientific exposure as the strongest positive correlates, and opposition concentrated in non-medical applications (33). In Malaysia, a survey among undergraduate students found substantially higher opposition to gene editing for enhancement compared to therapeutic use, with ethical reasoning often guided by principles such as necessity and harm prevention (34).

Consistent with this, regulatory governance has been identified as a key determinant of public confidence in these technologies; the absence of comprehensive legal frameworks amplifies uncertainty, whereas transparent biosafety oversight strengthens trust (35). Collectively, these findings suggest that the patterns observed in this study are not culturally idiosyncratic but reflect a consistent trend: therapeutic benefit and regulatory trust drive acceptance, while increased awareness may heighten ethical scrutiny rather than unconditional endorsement.

Overall, these findings suggest that public acceptance of therapeutic genome editing is shaped by a combination of perceived clinical benefit, sociocultural interpretation, and institutional trust. Understanding these factors is essential for guiding responsible clinical translation as genome editing technologies move closer to routine healthcare applications.

## 5 Strengths and limitations

This study represents one of the largest assessments of public attitudes toward genome editing conducted in Saudi Arabia and provides data from a region that remains underrepresented in the literature on genomic ethics and public perception. The questionnaire was adapted from previously published surveys and underwent expert validation and pilot testing. In addition, the study included participants from multiple geographic regions across the Kingdom and explored not only awareness and acceptance, but also psychological and contextual factors influencing attitudes toward therapeutic genome editing.

Several limitations should also be considered. First, the cross-sectional design limits causal interpretation of the observed associations between awareness, attitudes, and acceptance of genome editing technologies. Second, the online survey approach may have introduced recruitment and sampling bias, with overrepresentation of females, younger adults, highly educated individuals, and residents of the Central Region, potentially limiting generalizability to the broader Saudi population. Third, all responses were self-reported and may therefore be subject to recall, response, and social desirability bias. Finally, participants who were previously unfamiliar with genome editing were provided with a brief explanatory infographic before completing some survey items, which may have influenced subsequent responses.

## 6 Conclusion

This study provides a large-scale assessment of public awareness and acceptance of genome editing in Saudi Arabia. The strong overall support for therapeutic applications indicates a general receptiveness to clinical use, but with important cultural and contextual nuances. As genomic medicine continues to expand globally, these findings underscore the importance of inclusive policies, culturally responsive communication, and value sensitive education strategies. Incorporating perspectives from underrepresented regions, is essential for developing governance frameworks that are both globally relevant and socially responsive.

## Data Availability

The original contributions presented in the study are included in the article/Supplementary material, further inquiries can be directed to the corresponding author.

## References

[1] Jinek M Chylinski K Fonfara I Hauer M Doudna JA Charpentier E. A programmable dual-RNA-guided DNA endonuclease in adaptive bacterial immunity. Science. (2012) 337:816–21. doi: 10.1126/science.1225829, 22745249 PMC6286148

[2] Dunbar CE High KA Joung JK Kohn DB Ozawa K Sadelain M. Gene therapy comes of age. Science. (2018) 359:6372. doi: 10.1126/science.aan4672, 29326244

[3] Baltimore D Berg P Botchan M Carroll D Charo RA Church G . Biotechnology. A prudent path forward for genomic engineering and germline gene modification. Science. (2015) 348:36–8. doi: 10.1126/science.aab1028, 25791083 PMC4394183

[4] National Academies of Sciences E, Medicine, National Academy of M, National Academy of S. Committee on Human Gene Editing: Scientific M, Ethical C. Human Genome Editing: Science, Ethics, and Governance. Washington (DC): National Academies Press (US). 28796468

[5] Geuverink WP Houtman D Retel Helmrich IRA Kist JD Henneman L Cornel MC . A decade of public engagement regarding human germline gene editing: a systematic scoping review. Eur J Hum Genet. (2025) 33:570–9. doi: 10.1038/s41431-024-01740-6, 39609592 PMC12048525

[6] Conley JM Cadigan RJ Davis AM Juengst ET Kuczynski K Major R . The promise and reality of public engagement in the governance of human genome editing research. Am J Bioeth. (2023) 23:9–16. doi: 10.1080/15265161.2023.2207502, 37204137 PMC10367578

[7] Scheufele DA Xenos MA Howell EL Rose KM Brossard D Hardy BW. U.S. attitudes on human genome editing. Science. (2017) 357:553–4. doi: 10.1126/science.aan3708, 28798120

[8] Gaskell G Bard I Allansdottir A da Cunha RV Eduard P Hampel J . Public views on gene editing and its uses. Nat Biotechnol. (2017) 35:1021–3. doi: 10.1038/nbt.3958, 29121022

[9] Sawai T Hatta T Akatsuka K Fujita M. Human genome editing in clinical applications: Japanese lay and expert attitudes. Front Genet. (2023) 14:1205092. doi: 10.3389/fgene.2023.1205092, 37662845 PMC10469609

[10] Armsby AJ Bombard Y Garrison NA Halpern-Felsher BL Ormond KE. Attitudes of members of genetics professional societies toward human gene editing. CRISPR J. (2019) 2:331–9. doi: 10.1089/crispr.2019.0020, 31599688 PMC6791481

[11] Ramos PD Almeida MS Olsson IAS. What do people think about genetic engineering? A systematic review of questionnaire surveys before and after the introduction of CRISPR. Front Genome Ed. (2023) 5:1284547. doi: 10.3389/fgeed.2023.1284547, 38192431 PMC10773783

[12] Barlevy D Juengst E Kahn J Moreno J Lambert L Charo A . Governing with public engagement: an anticipatory approach to human genome editing. Sci Public Policy. (2024) 51:680–91. doi: 10.1093/scipol/scae010, 39035203 PMC11258878

[13] AlRasheed MM AlAli H Alsuwaid AF Khalaf S Ata SI BinDhim NF . Gene therapy knowledge and attitude among healthcare professionals: a cross-sectional study. Front Public Health. (2021) 9:773175. doi: 10.3389/fpubh.2021.773175, 34869185 PMC8634372

[14] Kobayashi S Miyoshi T Kobayashi T Hayakawa I Urayama KY Uchiyama M . Public attitudes in the clinical application of genome editing on human embryos in Japan: a cross-sectional survey across multiple stakeholders. J Hum Genet. (2022) 67:541–6. doi: 10.1038/s10038-022-01042-z, 35534678

[15] McCaughey T Sanfilippo PG Gooden GE Budden DM Fan L Fenwick E . A global social media survey of attitudes to human genome editing. Cell Stem Cell. (2016) 18:569–72. doi: 10.1016/j.stem.2016.04.011, 27152441

[16] Hair JF Black WC Babin BJ Anderson RE. Multivariate Data Analysis. New York: Pearson (2010). p. 785.

[17] Watanabe D Saito Y Tsuda M Ohsawa R. Increased awareness and decreased acceptance of genome-editing technology: the impact of the Chinese twin babies. PLoS One. (2020) 15:e0238128. doi: 10.1371/journal.pone.0238128, 32946484 PMC7500613

[18] McFadden BR Rumble JN Stofer KA Folta KM. U.S. public opinion about the safety of gene editing in the agriculture and medical fields and the amount of evidence needed to improve opinions. Front Bioeng. Biotechnol. (2024) 12:1340398. doi: 10.3389/fbioe.2024.1340398, 38433825 PMC10904643

[19] Oh SD Lee B. Analysis of the public perception and acceptance of gene-editing technology and gene-edited agricultural products in South Korea. GM Crops Food. (2025) 16:795–810. doi: 10.1080/21645698.2025.2576272, 41102994 PMC12536624

[20] Waltz M Juengst ET Edwards T Henderson GE Kuczynski KJ Conley JM . The view from the benches: scientists' perspectives on the uses and governance of human gene-editing research. Crispr j. (2021) 4:609–15. doi: 10.1089/crispr.2021.0038, 34406038 PMC8392077

[21] Jedwab A Vears DF Tse C Gyngell C. Genetics experience impacts attitudes towards germline gene editing: a survey of over 1500 members of the public. J Hum Genet. (2020) 65:1055–65. doi: 10.1038/s10038-020-0810-2, 32737393

[22] Shabana A. Between treatment and enhancement: Islamic discourses on the boundaries of human genetic modification. J Relig Ethics. (2022) 50:386–411. doi: 10.1111/jore.12404

[23] Alsomali N Hussein G. CRISPR-Cas9 and he Jiankui's case: an Islamic bioethics review using Maqasid al-shari'a and Qawaid Fighiyyah. Asian Bioeth Rev. (2021) 13:149–65. doi: 10.1007/s41649-021-00167-1, 34394752 PMC8298734

[24] Shineha R Takeda KF Yamaguchi Y Koizumi N. A comparative analysis of attitudes toward genome-edited food among Japanese public and scientific community. PLoS One. (2024) 19:e0300107. doi: 10.1371/journal.pone.0300107, 38625915 PMC11020778

[25] Unger JM Shulman LN Facktor MA Nelson H Fleury ME. National estimates of the participation of patients with Cancer in clinical research studies based on commission on Cancer accreditation data. J Clin Oncol. (2024) 42:2139–48. doi: 10.1200/JCO.23.01030, 38564681 PMC11191051

[26] Kubilay Tolunay P Erol C Kahraman S Yildiz Tacar S Özcan E Bugdayci Basal F . Understanding of clinical trials among patients with Cancer and their relatives. JAMA Netw Open. (2025) 8:e2457020. doi: 10.1001/jamanetworkopen.2024.57020, 39874033 PMC11775742

[27] Weisberg SM Badgio D Chatterjee A. A CRISPR New World: attitudes in the public toward innovations in human genetic modification. Front Public Health. (2017) 5:117. doi: 10.3389/fpubh.2017.00117, 28589120 PMC5439143

[28] Critchley C Nicol D Bruce G Walshe J Treleaven T Tuch B. Predicting public attitudes toward gene editing of germlines: the impact of moral and hereditary concern in human and animal applications. Front Genet. (2019) 9:704. doi: 10.3389/fgene.2018.00704, 30687386 PMC6334182

[29] Baum CM Kamrath C Bröring S De Steur H. Show me the benefits! Determinants of behavioral intentions towards CRISPR in the United States. Food Qual Prefer. (2023) 107:104842. doi: 10.1016/j.foodqual.2023.104842

[30] Secretariat SAV. Saudi Vision 2030. (2024). Available online at: https://www.vision2030.gov.sa/en/annual-reports

[31] Delhove J Osenk I Prichard I Donnelley M. Public acceptability of gene therapy and gene editing for human use: a systematic review. Hum Gene Ther. (2020) 31:20–46. doi: 10.1089/hum.2019.197, 31802714

[32] Abuhammad S Khabour OF Alzoubi KH. Researchers views about perceived harms and benefits of gene editing: a study from the MENA region. Heliyon. (2021) 7:e06860. doi: 10.1016/j.heliyon.2021.e06860, 33997394 PMC8095113

[33] Masoud AE Mesmar HA Omair M Alkaabi NM Alqaydi MM Dafalla MA . Knowledge, attitudes, and acceptance of genetic engineering among adults in the UAE: a cross-sectional study. Cureus. (2025) 17:e88365. doi: 10.7759/cureus.88365, 40842805 PMC12365708

[34] Muhsin SM Akbar MA Mustari S Alashaikh MH Chin AHB. Human cognitive enhancement and reprogenetic technologies in Malaysia—a survey study of local Muslim undergraduate students' viewpoints. Front Sociol. (2025) 10:1701007. doi: 10.3389/fsoc.2025.1701007, 41625625 PMC12853642

[35] Kalidasan V Theva DK. Is Malaysia ready for human gene editing: a regulatory, biosafety and biosecurity perspective. Front Bioeng Biotechnol. (2021) 9:649203. doi: 10.3389/fbioe.2021.649203, 33777918 PMC7992004

