# Supplementary material for: Public acceptance of genome editing in Saudi Arabia is driven by therapeutic benefit despite limited awareness
Source: Front Med (Lausanne). 2026 Jun 25;13:1827655. doi: 10.3389/fmed.2026.1827655 (PMC13345835; doi:10.3389/fmed.2026.1827655)
Supplement: Supplementary file 1 [file Data_Sheet_1.pdf]

Supplementary materials

Supplementary Figure 1.

Educational infographic presented to participants who were previously unfamiliar with genome editing. The infographic provided a simplified explanation of genome editing concepts and therapeutic applications before participants completed subsequent survey items.

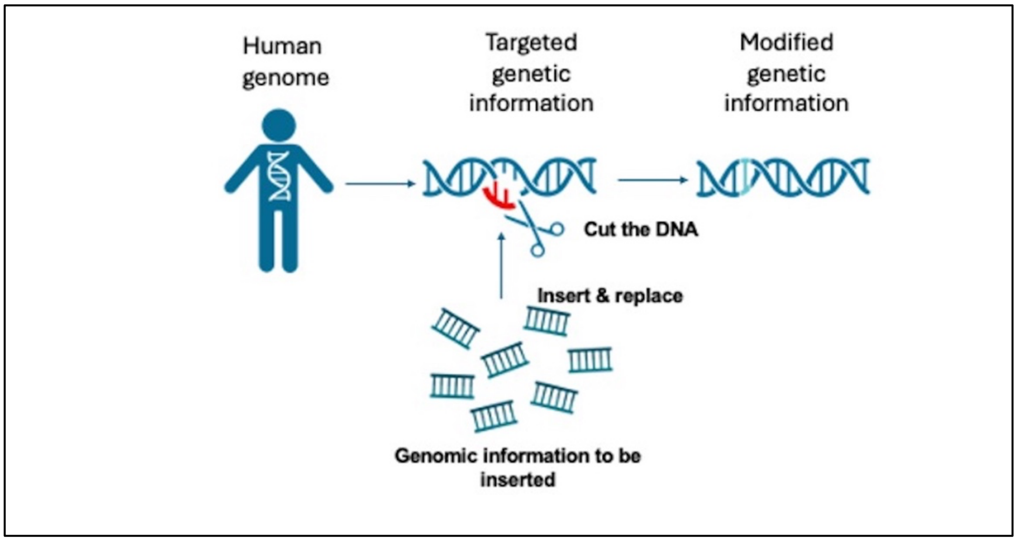

Supplementary Table 1. Acceptability of using genome editing.

Item	Strongly Disagree		Disagree		Neutral		Agree		Strongly Agree	
	N[a]	%	N	%	N	%	N	%	N	%
To treat blood cancer in patients who have no other treatment options	37	2.2%	33	1.9%	255	14.9%	638	37.3%	749	43.8%
In children or adults to treat life-threatening diseases	38	2.2%	56	3.3%	288	16.8%	678	39.6%	652	38.1%
To prevent life-threatening childhood-onset disease	62	3.6%	59	3.4%	299	17.5%	626	36.6%	666	38.9%
To prevent a debilitating disease	57	3.3%	76	4.4%	344	20.1%	596	34.8%	639	37.3%

[a] *Number of participants*
